# Unraveling the spectrum: overlap, distinctions, and nuances of ADHD and ASD in children

**DOI:** 10.3389/fpsyt.2024.1387179

**Published:** 2024-09-13

**Authors:** Sabrina Martinez, Kalin Stoyanov, Luis Carcache

**Affiliations:** Herbert Wertheim College of Medicine, Florida International University, Miami, FL, United States

**Keywords:** ADHD, ASD, autism, DSM, neurodevelopment, psychiatry, children, spectrum

## Abstract

This review explores the clinical presentation of similarities and differences in Attention-Deficit/Hyperactivity Disorder (ADHD) and Autism Spectrum Disorder (ASD). This paper investigates the deficits in executive function, social function, and emotional intelligence that are seen in both conditions and how the presence of both conditions can exacerbate these deficiencies. Understanding the clinical presentations in these domains is critical to refine diagnostic methods and treatments and improve outcomes for those affected by these neurodevelopmental disorders. The similarities in clinical presentation between ADHD and ASD present a significant diagnostic challenge, with individuals often exhibiting similar behaviors and difficulty navigating the complexities that encompass reacting to their environment. Further research is paramount in gaining more knowledge of the disorders and challenges faced by these individuals, especially those with the presence of both conditions.

## 1 Introduction

Autism Spectrum Disorder and Attention-Deficit/Hyperactivity Disorder are two neurodevelopmental disorders that initially present in childhood (1–3). ASD is a neurodevelopmental disorder that is distinguished by significant difficulties in social interactions and communication deficits in two or more settings when compared to neurotypical peers (1–3). Individuals with ASD also frequently exhibit restricted and repetitive behaviors, interests, and activities and may have difficulties with sensory processing. ADHD is a neurodevelopmental disorder that affects attention, hyperactivity, and impulsivity across multiple settings, leading to reduced cognitive or behavioral function when compared to neurotypical peers (1–4). The prevalence of ASD has steadily increased in recent years, with 2018 statistics noting that approximately 1 in every 44 children in the United States has ASD, or around 2.2% (1, 5). The prevalence of ADHD was estimated to be between 5-8% of children in the United States in 2013 (4). Additionally, ASD and ADHD have high rates of being co-diagnosed. Approximately 13% of children who have been diagnosed with ADHD will eventually also be diagnosed with co-occurring ASD (6). In children with ASD, there is a large amount of comorbid ADHD, with sources varying but generally ranging from 40-70% (6).

It is crucial to understand these two disorders, as their prevalence comprises a significant portion of children in the United States. Although at a cursory glance, the diagnostic criteria of ADHD and ASD appear to be very distinctive, the clinical presentations and manifestations of these two disorders may often lead to the disorders appearing similar in certain domains to one another. Deficits in executive function, social function, and emotional intelligence are seen in both conditions. This may lead to difficulty for clinicians when attempting to establish a diagnosis (7). The high rates of comorbidity among ADHD and ASD further add to the complexity of making an accurate diagnosis and formulating a treatment plan (3). In comorbid ADHD and ASD, the diagnosis of only one disorder frequently delays the diagnosis of a second comorbid condition, often by years (3). Making the correct diagnosis and extrapolating the correct pathology of an individual takes a keen eye and deep understanding of the subtle manifestations of these disorders, which will be discussed at length in this review paper.

## 2 DSM criteria change

The Diagnostic and Statistical Manual of Mental Disorders (DSM) is a standardized system used to classify and diagnose mental disorders, including neurodevelopmental disorders. The primary application of the DSM is clinical, allowing clinicians to understand the psychopathology that patients can face, come to a correct diagnosis, and apply this knowledge to patient management and treatment (2).

The most recent version of the DSM, the DSM-5-TR, was published in 2022. The DSM-4 had several key differences, which were changed in the DSM-5. The DSM-4 included “Pervasive Developmental Disorders,” which encompassed Autism, Asperger’s syndrome, Rett’s syndrome, and unspecified pervasive developmental disorders (2). The DSM-5 saw these diagnoses converge to different points on the same continuum, known as Autism Spectrum Disorder (2).

The DSM-4 categorized ADHD as one of several “Disorders Usually Diagnosed in Infancy, Childhood, and Adolescence.” The DSM-5 categorized ASD and ADHD in the same “family” of disorders, known as neurodevelopmental disorders, which has ultimately helped clinicians to deepen their understanding of these disorders and how these disorders affect and impact individuals not only in childhood but throughout their entire lives (2).

The changes in DSM-5-TR have decreased some of the difficulties clinicians encounter when making a diagnosis (2). One of the most significant changes that was made was that in the DSM-4, a comorbid diagnosis of ADHD and ASD could not be made (3, 4). Any inattentive or hyperactive/hyperkinetic symptoms that were present in a child with autism or a pervasive developmental disorder were thought to be due to the pervasive developmental disorder, thus making the presence of a pervasive developmental disorder an exclusionary criterion to the diagnosis of ADHD. Clinicians are now able to make a comorbid diagnosis of ASD and ADHD through the DSM-5-TR (2, 8).

Although diagnostic criteria have evolved from DSM-4 to DSM-5-TR, the real-world clinical application remains challenging due to the limited studies directly comparing ASD, ADHD, and their comorbid presentation.

## 3 Areas of impacted functioning

### 3.1 Executive function

One of the main areas of deficits seen in individuals with ADHD and ASD, when compared to neurotypical peers, is deficits in executive function (9). Executive function consists of the cognitive skills that allow individuals to self-regulate and perform self-directed behaviors, enabling them to plan, execute ideas, and achieve goals (3, 7, 10, 11). Executive function also allows inhibition of one’s responses and permits cognitive flexibility (3, 7, 11). Cognitive flexibility is a critical component of daily functioning, as it describes an individual’s ability to adapt and react to novel stimuli and a changing environment (10, 12–14). Executive function is also responsible for working memory, which is the ability to retain and manipulate new information (3, 7, 10, 11, 15).

**Figure 1:**
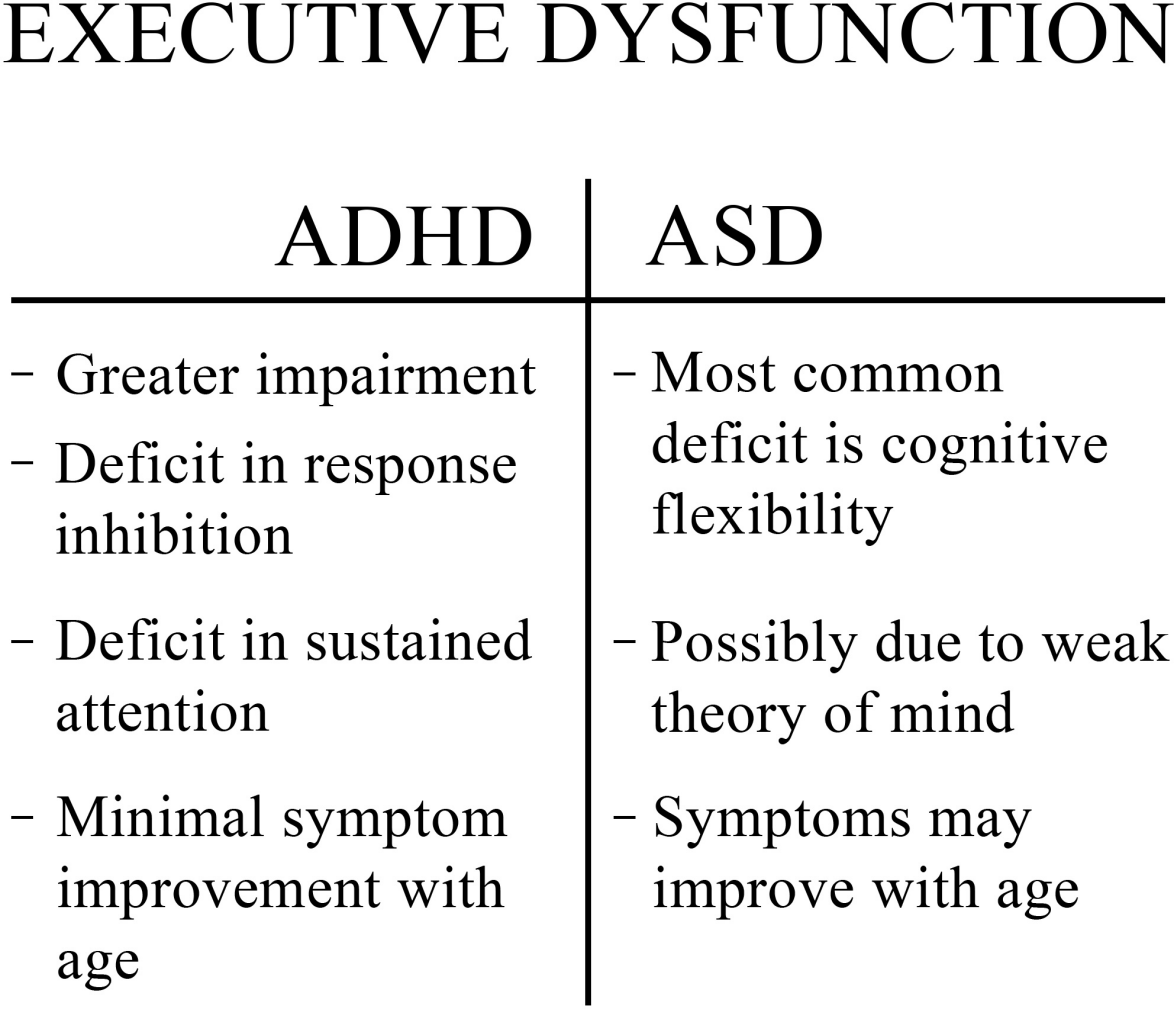
Comparison of executive dysfunction in ADHD and ASD.

Deficits in executive function have been noticed in the profiles of children as young as preschoolers who have ASD or ADHD (16). While ADHD and ASD both have deficits in executive functioning, the exact areas in which each disorder is typically lacking differ, giving them each a unique “profile” (5, 7). However, executive function is generally worse in ADHD (3, 5). Due to difficulties with inhibition and sustained attention, children become hyperactive and inattentive, which are the core features of ADHD (**Figure 1**) (7, 10, 17). Poor regulation of inhibition additionally results in increased impulsivity (10). Those with ADHD may also have challenges with innovation, planning, and problem-solving (3). Another distinction to note is that executive dysfunction in ASD generally has shown improvements with age; in contrast, executive dysfunction in ADHD generally shows minimal improvement with age (3).

Although individuals with ADHD have more severe impairments in executive functioning, the deficits seen in those with ASD are not less significant. For example, individuals with ASD often have diminished perseverance when compared to their peers, especially when planning and shifting between tasks (7). The most common executive dysfunction in ASD is a lack of cognitive flexibility (**Figure 1**). Deficient cognitive flexibility leads to problems adapting to changes in routine or environment, problems alternating between tasks and fixed and repetitive behaviors and interests (10, 14).

Some studies, however, have hypothesized that the executive dysfunction seen in ASD may be caused by poor high-level cognition and an inability to infer (10, 18). Difficulties with inference can lead to a poor theory of mind, which is the ability to understand and predict the mental states and actions of other individuals (10, 18–20). Executive function has a strong association with theory of mind. Thus, a poorly developed theory of mind could be perceived as a confounder that has perpetuated the idea that executive dysfunction is the cause of poor functioning in ASD, even though the primary deficit may have always been a deficit in theory of mind (18, 19). This alternative mechanism should be considered, as research has shown that some individuals with ASD without a co-occurring intellectual disability are strong in several regions of executive functioning, such as memory and inhibition (10). However, these individuals often still struggle in other areas of executive functioning- namely cognitive flexibility and planning ability (10). A comparison of the executive dysfunction in ADHD and ASD can be seen in **Figure 1**.

When considering executive dysfunction, clinicians should more carefully consider their impacts on those with co-occurring disorders (10). A systematic review of executive function deficits in children with comorbid conditions elucidated studies that tracked performance on indirect assessments and performance-based assessments. Performance-based investigations are often performed in highly controlled clinical settings and do not always reflect real-world function (pure problem-solving). In contrast, indirect assessments show executive functioning (problem-solving and adaptability) in real-world settings (13). In performance-based assessments, those with comorbid diagnoses performed more poorly in flexibility, shifting, and attention than either diagnosis alone (13). However, results in inhibition, organization, planning, and working memory were similar to single-diagnosis counterparts (13). In indirect assessments, those with comorbid diagnoses performed more poorly in inhibition and inattention, with similar results in organization and planning (13). In comparison, a 2024 meta-analysis assessed 36 studies that compare executive function in children with ADHD, ASD, comorbid ADHD and ASD, and compared them with neurotypical children (9). The authors used performance-based neuropsychological tests as a proxy assessment of high-order cognitive processes. While limitations existed due to a widespread lack of studies focusing on co-occurring ADHD and ASD, this group was found to be most similar to the ADHD-only group and worse than the ASD-only group in neuropsychological testing in one included study (9).

⇨ Key Point:

While ADHD and ASD are both associated with executive function deficits, the areas of impairment differ. ADHD primarily impacts inhibition and sustained attention, leading to hyperactivity and inattention, while ASD is more associated with deficits in cognitive flexibility.

⇨ Key Point:

Individuals with both ADHD and ASD may exhibit worse executive function impairments than those with either condition alone.

### 3.2 Social functioning

**Figure 2:**
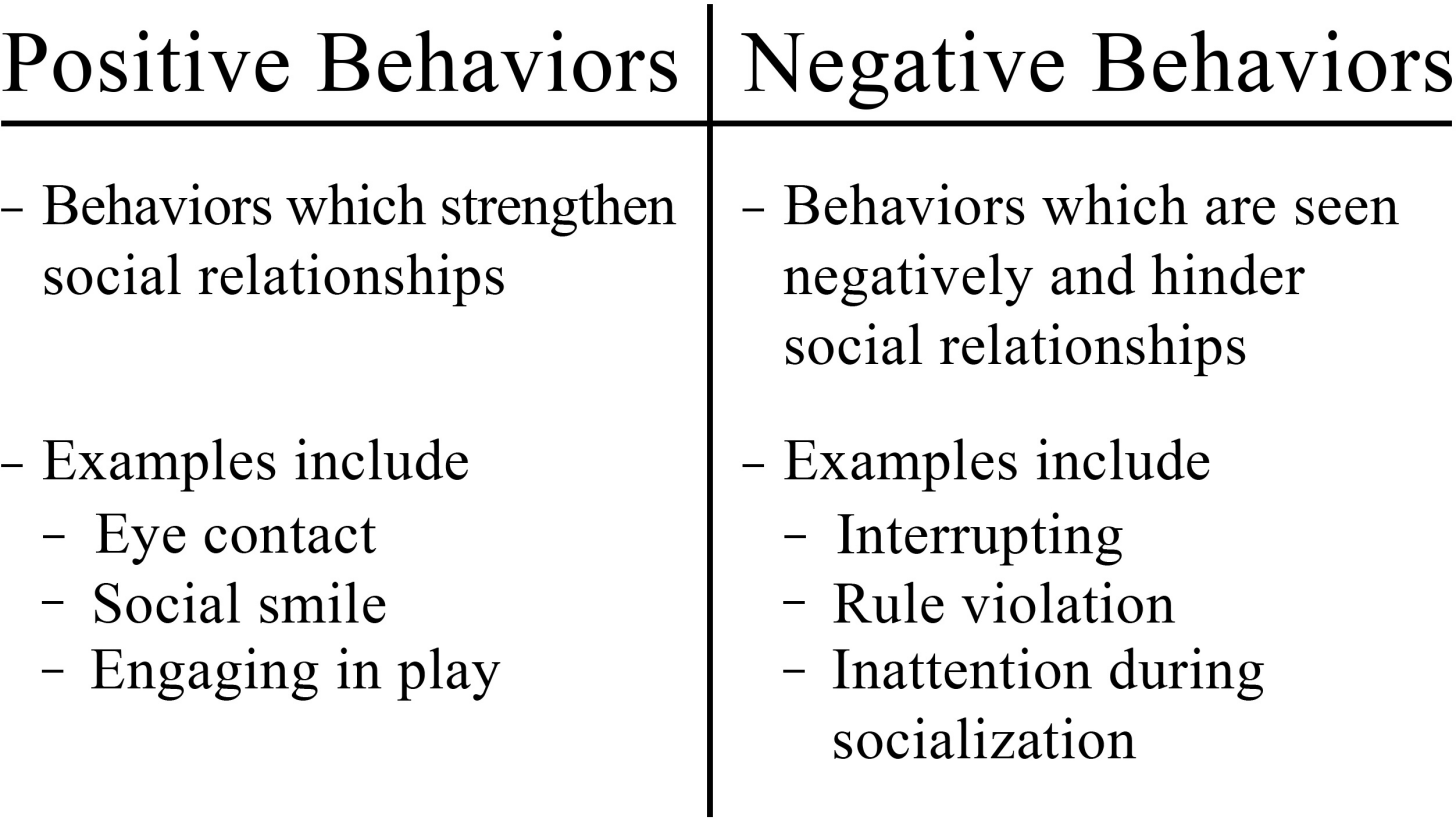
Comparison of positive and negative behaviors.

Social functioning allows individuals to function interdependently in society. Similarly to how executive dysfunction was the hallmark of ADHD, social dysfunction encompasses a large portion of the core deficits in ASD. Many factors separate and hinder individuals with ASD from appropriate socialization with their peers. One set of factors is a lack of behaviors that are seen positively and strengthen social relationships (**Figure 2**). For example, children with ASD often don’t make enough eye contact or display joint or shared attention with their peers (21, 22). Difficulties in executive function, such as focusing attention, impair social relationships (15). Children with ASD frequently lack a social smile, attention, and shared enjoyment of different activities or topics due to their restricted interests (6, 21). Thus, they fail to respond appropriately to peers during social interactions (21). These difficulties result in a lack of perceived empathy, leading to problems choosing and navigating different conversational topics (21) The lack of skills and positive behaviors needed to socialize with peers culminates in noticeable deficits that impair even basic social interactions (21, 22). Thus, significant difficulties are encountered with complex social interactions and interpreting social contexts and cues (6, 21). Additionally, children with ASD not only lack the skills to play and make friends with peers but often lack the interest in establishing friendships altogether. Many individuals with autism prefer to do things alone, often withdrawing from their peers and avoiding social interactions (21).

**Figure 3:**
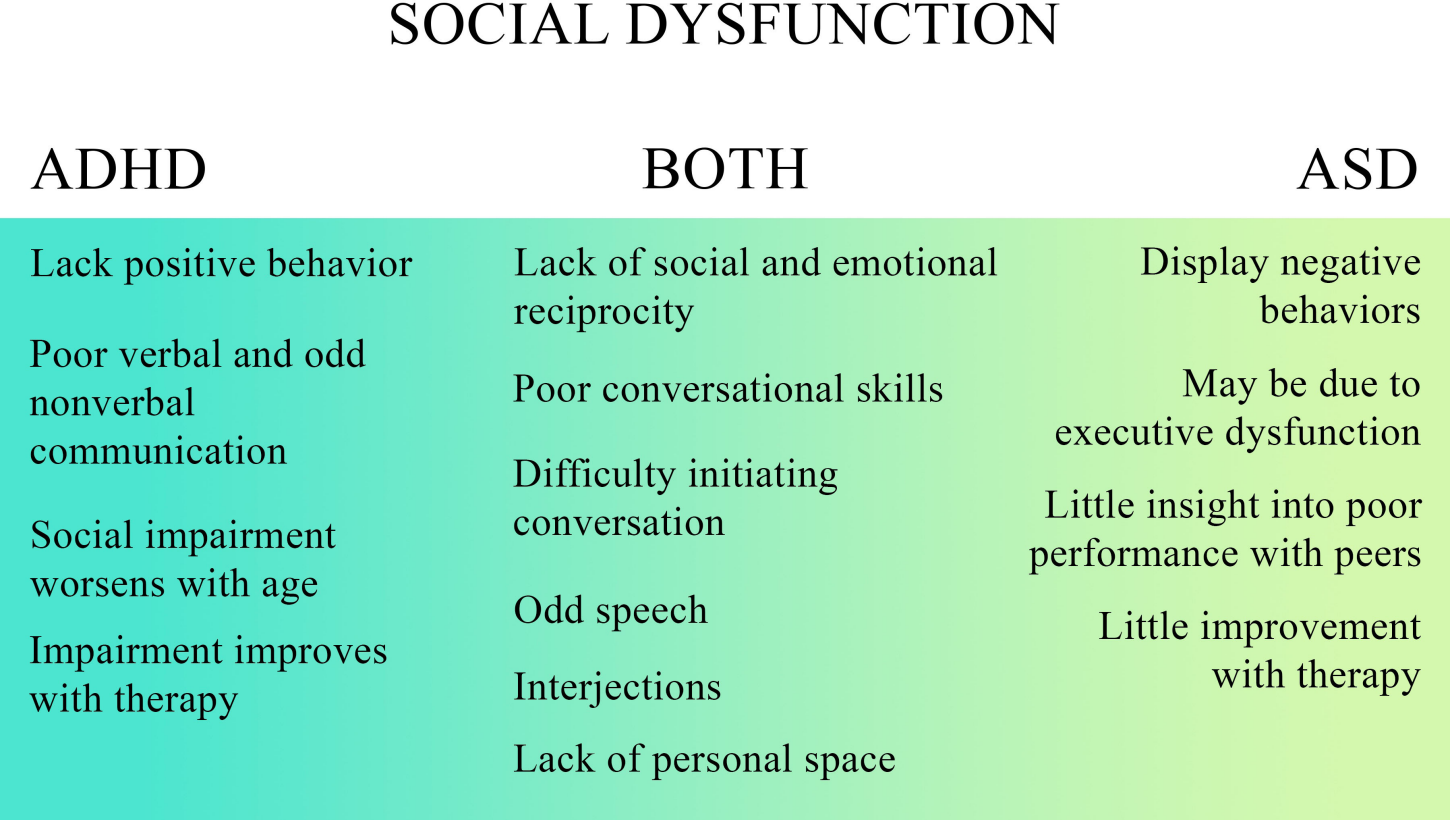
Comparison of social behaviors in ADHD and ASD.

Children with ASD may also have an atypical approach to social situations, and the oddities they display lead to poor communication, both verbal and nonverbal (**Figure 3**). Communication differences may include unusual production of speech, peculiar speech patterns, odd vocal intonations and inflections, and difficulties with paralinguistics (23). Difficulties with paralinguistics impair the reciprocity commonly seen in conversation and make it more difficult for these children to develop and maintain friendships beyond childhood (23). Individuals with ASD additionally often have strange body language and unusual mannerisms (21). Their peers may see repetitive and self-soothing behaviors, such as body rocking, vocal noises, hand or finger movements, or flapping movements as atypical, leading to exclusion from groups (1, 23). Their touches may also appear intrusive to their peers (21). Of note, these difficulties worsen with age as the difference between their expected and actual social functioning continues to widen with time (**Figure 3**) (23). Therefore, while children with ASD can sometimes develop friendships when they are young, and peers are more forgiving, this becomes increasingly difficult when they are unable to overcome the continued increase in expectations for social relations (23).

While social impairment is not required as part of the diagnosis for ADHD, it is often present (3). Social impairments in ADHD may be due to either a central social dysfunction or the result of executive dysfunction, particularly lack of attention and inhibition, causing impulsivity and hyperactivity (3, 6). For example, inattention can cause social cues to be missed, and impulsivity can upset other children (21). Further, hyperactivity can cause decreased participation in social events (21). Those with ADHD also face difficulties with theory of mind, although far less severe than is seen in ASD (24). Other common complaints may include “often has difficulty waiting in line,” “often blurts out answers,” and “often interrupts or intrudes on others,” as seen in ADHD diagnostic questionnaires; parents commonly note that these habits cause social difficulties (3). Other socially inappropriate behaviors often noted are intrusiveness, impulsive speech, and impulsive aggressive behaviors (5, 6). Specific behaviors include being “annoying,” controlling, explosive, not paying attention during activities, and rule violations (**Figure 2**) (21).

Children with ADHD often have very little insight into their social skills and functioning, leading to interpersonal dysfunction and difficulties with their relationships (**Figure 3**) (6). Poor social functioning may lead children with ADHD to be rejected by their peers more often, thus developing a “negative reputation” with peers. The impact of this negative reputation can last even after receiving interventions and treatment to enhance social skills; peer denial is still present, even with measurable improvement in social skills (6, 21).

A critical difference between those with ADHD and ASD is that children with ADHD have the social knowledge that they need to make friendships and develop socially (3). However, they have a “poor performance” when interacting with peers (3). So, although individuals with ADHD might know what to do, they are often unable to execute socially correct behaviors (3). Conversely, children with ASD have social knowledge deficits and don’t understand how or have the skills needed to make friends (3). Thus, they do not perform well with their peers due to their knowledge gap (3). When children with ASD receive clinical social skills and friendship development training, individuals with ASD are much more likely to show improvement in social skills when compared to children with ADHD (3). Children with ADHD generally show less improvement in social skills after training and display less growth and change.

Some studies have found significant similarities in social deficits between ADHD and ASD, most often in the lack of social and emotional reciprocation, poor conversational skills, restricted interests, rituals, stereotypes, and preoccupations (25–27). Many parents of children with ADHD have noted difficulties in social functioning which are similar to those in autism and autism-like symptoms; symptoms described include difficulties with holding conversations (especially initiating conversations), odd speech patterns, irrelevant interjections, stereotyped movements, and issues with understanding and participating in nonverbal communication, including respecting personal space boundaries (25). A comparison of similarities and differences in social dysfunction in ADHD and ASD can be seen in **Figure 3**.

Few studies have been done on the effect of comorbid ASD and ADHD on social functioning. Previous studies by Rao in 2014 and Factor in 2017 found that children with comorbid ADHD and ASD had significantly greater social impairment compared to their ASD-only counterparts (28, 29). Both of these studies utilized the Social Responsiveness Scale, 2nd Edition (SRS-2), an autism assessment completed by parents or teachers that is designed to assess the severity of social impairment in ASD (28, 29). However, a 2015 study completed by Salley revealed that individuals with ASD-only had worse social impairment than comorbid ASD and ADHD groups. The study completed by Salley utilized the Autism Diagnostic Observation Schedule (ADOS), a standardized assessment completed by trained clinicians to evaluate several skills, including social interaction, in individuals with ASD (30). A more recent study by Harkins in 2022 compared patients using the standardized ADOS-2 and found no significant differences in the comorbid ASD and ADHD group when compared to the ASD-only group (21). The differences between these studies may be explained by differences in the study groups and measurement tools used between studies. While Rao and Factor studied individuals in the community, Salley and Harkins studied individuals who had been referred clinically and may have had more severe symptoms of ASD as compared to individuals in the community (21, 28–30). Furthermore, Rao and Factor utilized the SRS-2 assessment, which is completed by parents who may overestimate certain behaviors, while Salley and Harkins utilized the ADOS assessment, which is observational and completed by clinicians who may underestimate certain behaviors (21, 28–30).

Additionally, the authors in the Salley 2015 study noted that there was a different distribution of the ADOS modules in the comorbid ASD and ADHD group when compared to the module distribution in all of the other groups (30). Thus, there is the potential that the scores for this group were suppressed (30). Although results from studies throughout the years have been inconsistent, it appears that studies using objective measures of data in a controlled environment seem to favor the idea that a comorbid diagnosis does not worsen social functioning when compared to ASD-only groups. However, the SRS-2 assessment may be more favorable in measuring global social functioning, in addition to the fact that the ADOS is targeted towards assessing features of autism, which may overlook social impairments attributable to ADHD (21).

⇨ Key Point:

While children with ADHD often know what to do but fail to execute, children with ASD typically lack the social knowledge entirely. Children with ASD may benefit more from social skills training than those with ADHD, likely due to the difference between social knowledge and social performance deficits.

⇨ Key Point:

Research on the social functioning of individuals with comorbid ASD and ADHD is mixed. Some studies suggest greater social impairment in comorbid cases compared to ASD alone, while others find no significant difference. Differences in study populations and measurement tools may account for some of these inconsistencies.

### 3.3 Emotional intelligence

The differences and overlap of emotional intelligence in ADHD and ASD are complex. One part of emotional intelligence is the ability to recognize emotions. It has been long known that children with ASD have deficits in emotion recognition, possibly due to a central impairment and lack of social cognition; studies have delved into the reasons behind these inabilities to recognize emotions, such as lack of eye gaze and decreased visual attention (3, 7, 20). One test that suggests a central dysfunction is the reading of the “eyes test,” where an individual looks at a picture of eyes displaying different facial expressions in black and white and then selects the emotion being conveyed from a set of options (3). While those with ASD have the lowest score, or most significant deficits in social perception and cognition, those with ADHD have an intermediate score, showing deficits in both disorders (3). Individuals with ADHD perform most poorly when identifying anger and fear (7, 24). Not only do children with ASD have a decreased ability to recognize emotions, but they also do not have the same autonomic responses, such as variations in heart rate and respiration rate, in response to another person’s emotional expressions that neurotypical individuals exhibit (7). Similarly to social impairments, difficulties in emotion recognition may increase with time, thus widening the gap between those with ASD and neurotypical peers (20, 24).

Emotion regulation is the individual’s ability to modulate or modify the actual occurrence and degree of their emotions (23, 26). Emotion dysregulation impacts the ability to cope and function in society due to its association with poor social skills and acceptance by peers (23, 26, 27).

Children with ADHD have difficulties regulating their emotions, with studies showing that around 50% exhibit emotion dysregulation (23, 26, 27). It is difficult to ascertain the degree of emotional dysregulation that children with ASD possess because emotional dysregulation may be masked, overlooked, or conflated with the diagnostic criteria of the disorder. Essentially, emotional dysregulation may become “lumped in” with core diagnostic features of ASD, and thus, emotional problems tangential to the actual diagnostic criteria may be overlooked. For example, a clinician may see a child experiencing a tantrum and mistakenly assume that this is a feature of ASD rather than a separate problem with emotional regulation (23).

## 4 Masquerading symptoms

Despite the differences discussed in executive, social, and emotional domains, core features of either disorder may masquerade as symptoms more commonly seen in the other disorder.

**Figure 4:**
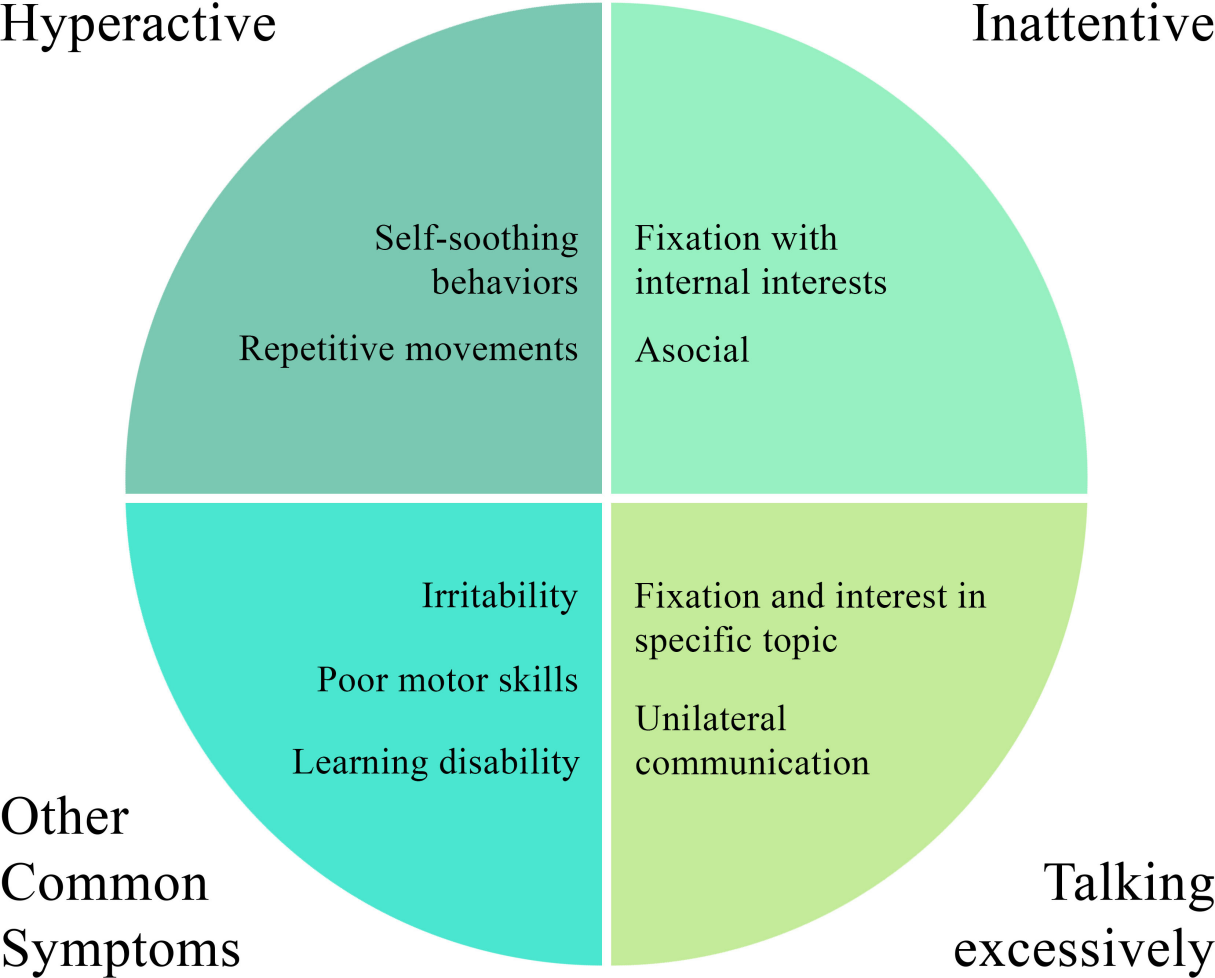
Explanations for common clinical features in ASD and ADHD.

For example, while children with ASD may truly have inattention and hyperactivity due to a secondary diagnosis of ADHD, children with ASD often exhibit symptoms of inattention or hyperactivity even if they do not have an additional diagnosis of ADHD. This may be due to core features of ASD appearing as ADHD-like symptoms (6). Upon closer investigation, the “inattention”-like symptoms seen in some children with ASD may just be due to their disinterest in social ongoings and behaviors and preoccupation with their own fixated and internal interests (**Figure 4**) (6). Therefore, while a child with ASD may pay close attention to an inanimate object or even a thought, they may appear to be “inattentive” to the social situations at hand (6).

Similarly, ADHD questionnaires often contain a statement along the lines of “this child may often seem not to listen when spoken to directly” (31). A similar complaint is often voiced by parents or teachers of children with ASD; however, these behavioral issues may not always be due to a true deficit in sustained attention, but once again may be due to the specific interests and internal fixation of those with ASD (1, 31). Similarly, due to their restricted interests, if a conversation arises regarding a topic that interests them, they may frequently “talk excessively” or “interrupt and intrude on others,” which are additional ADHD clinical features (**Figure 4**) (31). This is likely due to a fixated and extreme interest in one subject combined with poor social and communication skills (31). Children with autism often exhibit unilateral communication (talking “at” one person) rather than reciprocal communication in the usual back-and-forth manner (31).

Another example is that, “hyperactivity” symptoms, such as fidgeting, may be noticed due to the repetitive movements children with autism often perform (**Figure 4**) (6). These movements may intensify in new situations or unfamiliar settings or if an individual with ASD is feeling agitated (6). Thus, these repetitive movements may appear as fidgeting and hyperactivity when they are actually a self-soothing mechanism and based on a core feature of ASD. When inattention and hyperactivity present in this way in children with ASD, an additional diagnosis of ADHD may be unnecessary (6).

There is a wide range of symptoms that individuals with ASD or ADHD display more frequently (**Figure 4**) (25). Individuals with ASD or ADHD often have higher levels of anger, irritability, and general behavioral issues (25). Both groups also exhibit dysgraphia, slow processing speed, and learning disabilities- especially in written formats- and thus have language delays (25). They often experience deficits in perception of the world around them (25). Studies have also found significant differences between ADHD and ASD in the areas of communication deficits, restricted and repetitive behaviors and movements, difficulties in adapting to change, and reaction to sound and sensory input (25). Both groups exhibit deficits in executive functioning. Although not officially part of either diagnostic criterion, difficulties and deficits in motor coordination and motor persistence are often seen in ADHD and ASD (6). Children with ADHD may additionally show difficulty in inhibiting motor responses, possibly contributing to hyperactivity (6). **Figure 4** offers an overview of the common clinical features seen in ASD and ADHD.

⇨ Key Point:

Clinicians often struggle to distinguish ADHD from ASD due to the possibility that the core features of one disorder may appear similar to behaviors typically seen in the other disorder.

## 5 Presenting symptoms in co-occurring ADHD and ASD

The final, and least studied, issue is that of co-occurring ADHD and ASD. Individuals with co-occurring ADHD and ASD diagnoses face a large variety of challenges. In children with comorbid ADHD and ASD, the diagnosis of ADHD sometimes precedes the diagnosis of ASD (17, 25). This may be due to the fact that ADHD symptoms may sometimes cause noticeable functional difficulty for children sooner than symptoms of autism (17). ADHD is often diagnosed in elementary or middle school when the demands of school and an increased number of social interactions unveil core deficiencies (17). After ADHD symptoms are managed with medication, further behavioral and social problems due to comorbid ASD may become unmasked and apparent (1, 32).

In children with co-occurring ASD and ADHD, one study found inattention symptoms of ADHD to be most prevalent, found in 46% of children with ASD, 22% had primarily hyperactivity symptoms, and 32% had prevalent symptoms of both inattention and hyperactivity (4).

One study found that there are specific symptoms of ADHD that have been shown to have a relatively strong association with co-occurring symptoms of autism (31). These symptoms are when a child “does not wait for a turn,” “intrudes on others,” “talks excessively,” “does not sustain attention,” or “fidgets hands or feet; squirms in seat” (31). These symptoms should alert clinicians to more carefully consider co-occurring ADHD and ASD due to this association (31). However, a similar significance could not be given to any symptoms of autism (31).

Generally speaking, ASD is principally considered to be a more “severe” disorder due to generally higher degrees of impairment and functional difficulties (6). However, it has been noted that individuals with ASD who meet the full diagnostic criteria for ADHD often face more severe functional impairment than individuals with ASD who present with fewer symptoms of ADHD (6). Children with a comorbid diagnosis had poorer communication and social skills, poor adaptive functioning, and less adequate daily living skills (6, 33, 34).

A comorbid diagnosis may also intensify the symptoms of ASD or ADHD to greater severities than when either diagnosis occurs alone. For example, there is reduced emotion recognition in children with co-occurring ASD and ADHD (34). Furthermore, those with overlapping ASD and ADHD report having more symptoms of ASD than individuals with ASD alone (33). Not only is there an increased number of symptoms of autism in individuals with a comorbid diagnosis, but the symptoms are more strongly expressed (33). Additionally, attention problems are also worse in those with a comorbid diagnosis compared to those with ADHD alone, thus indicating that the presence of ASD may also worsen the symptoms of ADHD (34).

Those with overlapping disorders were found to be more likely to externalize issues into behavioral problems, including more tantrum-like behaviors (5, 33, 34). Finally, children with a comorbid diagnosis are also more likely to face other psychological issues, such as conduct problems, anxiety, or depression (34). Thus, overall, co-occurring ADHD and ASD is somewhat synergistic and is generally recognized to result in a more severe clinical symptomatology and lower quality of life than either disorder alone, illustrating the need to be more careful in providing well-thought-out treatment to these patients (34).

⇨ Key Point:

Individuals with comorbid ADHD and ASD typically display more severe symptoms than those with just one condition. Symptoms such as inattention and hyperactivity are often more pronounced, leading to greater challenges in areas like communication, social skills, and adaptive functioning.

⇨ Key Point:

The combination of ASD and ADHD usually results in more severe functional impairments and a lower quality of life.

## 6 Treatments and interventions

Gaining a thorough understanding of the subtleties of ADHD and ASD are imperative in order to guide proper treatment. The long-term consequences of not treating these conditions have been studied in the literature. ADHD persists into adulthood in around half of children diagnosed with ADHD (35). The most well-studied consequences of untreated ADHD in adulthood are substance use disorder and antisocial behaviors, often leading to incarceration (35, 36). Other consequences of lack of treatment are poorer physical and mental health, including increased inpatient psychiatric admissions (36). Unaddressed deficits in executive functioning also impair academic performance, pursuit of higher education, and ultimately may cause difficulties with work (36, 37).

Conversely, treatment of these disorders leads to improved outcomes in the lives of treated individuals. For example, a systematic review in 2022 found that evidence-based treatment of individuals with ADHD with stimulants led to improvements in cognitive function, such as in memory, attention, and, ultimately, academic performance (38). The improvement in cognition led to a subsequent improvement in the quality of life of individuals with ADHD (38). Improvements in executive function and working memory have also been seen across studies (38–40).

The mainstay of treatment for ADHD currently consists of stimulant medications or noradrenergic agonists (38, 41). Stimulant medications, such as methylphenidate, increase levels of dopamine and norepinephrine in the brain through the blockade of dopamine transporters (38, 41). Noradrenergic agonists, such as atomoxetine, increase the activity of both noradrenaline and dopamine in the brain (41). Two systematic reviews showed that treatment of ADHD with stimulants resulted in reduced symptoms of ADHD and improvements in cognitive and executive function, including in areas of attention, memory, and inhibition (38, 41). Although medications such as atomoxetine showed improvements in many areas of executive function, such as attention and inhibition, there were non-significant improvements in working memory (41). Physical activity interventions can also be considered as a complement to pharmacological interventions for children with ADHD. Multiple meta-analyses in recent years have also demonstrated the improvement of core features, such as inattention and inhibition, cognitive flexibility, and executive function in ADHD (42–47). This information is useful to help clinicians decide on the correct intervention strategy, depending on the tolerability of medications, patient and parents’ desires, and executive function profile of the patient.

The treatment of ASD is complex. In ASD, community and intensive behavioral interventions have been shown to improve adaptive behaviors and improve outcomes (40). Furthermore, interventions for executive function seem to show “far effects”, where improvements in skills or behaviors not explicitly trained are seen, showing that skills are able to be transferred (48). Current treatment is often targeted to help control undesirable behaviors such as irritability, aggression, anxiety, sleep disturbances, self-harm, and bothersome repetitive behaviors (49). Applied behavioral analysis (ABA) is the first line for behavioral treatment in ASD and has been shown by a 2023 meta-analysis to improve intellectual functioning and adaptive behaviors (50). While first-line treatment of ASD generally includes ABA, pharmacological treatments such as atypical antipsychotics can be used as adjuvants for symptomatic control (49). Aripiprazole and risperidone are currently the only antipsychotic drugs approved by the Food and Drug Administration to treat irritability in ASD (49, 51). Atypical antipsychotics are useful in managing poor impulse control, aggression, and hyperactivity (49, 51). Other medications often used include selective serotonin reuptake inhibitors (SSRIs) and buspirone, which are used to manage restrictive and repetitive behaviors (49). SSRIs may additionally help reduce aggressive and irritable behaviors (49). Due to the significant level of comorbid ADHD and ASD, ADHD symptoms in patients with ASD-only or a comorbid diagnosis can be treated with amphetamines, methylphenidate, atomoxetine, guanfacine, or viloxazine (52, 53). Clonidine may also be used (49). Generally, while these medications show improvement in attention and inhibition, they are less efficacious for managing symptoms of inattention, impulsivity, and hyperactivity in comorbid ASD and ADHD than in ADHD alone (49, 53).

Repetitive Transcranial Magnetic Stimulation (rTMS) has been investigated for ADHD, ASD, and co-occurring ADHD and ASD, with mixed results. For ADHD, studies suggest rTMS targeting the dorsolateral prefrontal cortex (DLPFC) may improve attention and executive functioning, however, larger trials are needed to confirm efficacy (54–56). In ASD, rTMS has shown more consistent benefits. Targeting the DLPFC has improved cognitive control, attention, and task-relevant discrimination (54). Low-frequency rTMS particularly improves behavioral outcomes, including better social interaction and reduced repetitive behaviors (57, 58). In ASD, rTMS is noted as an alternative for patients who are not suitable for psychopharmacological treatments (57). For co-occurring ADHD and ASD, research is still at a preliminary stage. The rTMS effects would, of course, vary depending on the brain regions stimulated. Depending on the configuration, DLPFC stimulation might improve ADHD-related attention, its impact on ASD symptoms, such as social cognition and behavioral regulation, remains less clear and requires further investigation. Another future direction for treatment of ADHD and ASD could include transcranial direct current stimulation (tDCS) (59, 60). A systematic review studying the effects of tDCS in ADHD and ASD was found to be at least partially effective, opening an avenue for further research in development of efficacious treatment modalities (59).

## 7 Discussion

This review gives an updated current overview of understanding the characteristics of ASD and ADHD following updates in diagnostic criteria in the DSM-5. It also displays the challenges faced by clinicians today when considering comorbid ADHD and ASD diagnoses. The manuscript explores subtleties in presentation and outlines models that provide useful frameworks for contextualizing the behaviors of patients with these conditions. Additionally, this manuscript highlights the need for accurate diagnosis for superior treatment results and improvement in patients’ functional status.

The significant prevalence of ADHD and ASD, which continues to increase, underscores the importance of understanding their clinical symptomatology. The release of the DSM-5 saw several changes, including that ADHD and ASD are no longer mutually exclusive diagnoses. Subsequently, research has shown that the rates of co-occurring diagnoses are high. In research performed before the criteria changed, children with overlapping symptoms were often excluded or poorly accounted for. After the criterion changed, there has been an increase in literature comparing the two disorders and analyzing individuals with comorbid diagnoses. However, there still has not been enough, and many studies that have come out have had conflicting results, making it difficult to come to well-supported conclusions regarding symptomatology and treatment options. Throughout a thorough review of the literature, authors often noted limitations in interpretation. In situations where comorbid ADHD and ASD groups were analyzed, there was difficulty in drawing significant conclusions as the number of studies focusing on this group was limited. In studies that analyzed ADHD-only versus ASD-only, results may be inaccurate because there is an unknown number of comorbid diagnoses that could have impacted results. Therefore, increasing research efforts specifically analyzing individuals with both diagnoses is imperative.

Not only has scientific research not sufficiently caught up to clinical decision-making, but another practical clinical consideration that makes diagnosis challenging is that many practicing clinicians are accustomed to thinking about these conditions as discrete pathologies that may not overlap. Introducing subtlety in adding a third diagnostic option, comorbid ADHD and ASD, may add unforeseen complexity to the decision-making model.

There are further challenges that clinicians need to take into account when considering a proper diagnosis. Since ADHD and ASD often present with similar behaviors, a challenge is posed for clinicians to distinguish the two conditions when one is present since both disorders share deficits in executive function, social function, and emotional intelligence. Although ASD is thought of as a more severe disorder because of its ability to limit an individual’s functional capacity, ADHD may be diagnosed first due if there are prominent deficits in executive functioning, delaying the correct diagnosis. These deficits become very apparent in school-age children and cause various issues, from achievement in school to interactions with peers. Thus, deficits in executive functioning can impact a child’s academic performance and ultimately have life-long consequences. The most significant deficits in executive function seen in ADHD are in inhibition and sustained attention, which cause the majority of the dysfunction and core features seen in ADHD as compared to neurotypical children. The most consistent and prominent deficit in executive function in individuals with ASD is in cognitive flexibility, leading to many of the restrictive and repetitive thoughts and patterns of behavior, as well as the difficulty seen with adapting to any changes outside of their expected routine. A co-occurring diagnosis of ADHD and ASD often leads to worse executive function. Mixed symptoms require clinicians to meticulously differentiate between ADHD’s attention deficits and ASD’s cognitive rigidity, necessitating thorough evaluations from multiple sources. When comparing the differences in executive function, it becomes apparent that having a deep understanding and keen eye for examining deficits in executive function can help clinicians distinguish between diagnoses and consider a comorbid diagnosis in an individual.

Children with ADHD display behaviors that are often socially inappropriate, such as interrupting or not waiting their turn, causing a poor reputation with their peers. The poor interpersonal relationships caused by negative, unsocial behaviors are often poorly understood by the individual with ADHD. The social dysfunction seen in children with ASD is complex and multifaceted. One component is a lack of positive, prosocial behaviors, such as eye contact and shared interests. Another component is odd behaviors and poor nonverbal communication, such as strange mannerisms, speech patterns, and self-soothing behaviors. Without intervention, their poor social performance increasingly isolates them from their peers as they age. However, with intervention, children with ASD show more improvement in social and executive functioning than ADHD peers. Difficulties with emotion regulation and recognition are seen in both disorders, which may hinder societal functioning and successful engagement in complex social interactions. Once again, these difficulties are magnified in those with co-occurring diagnoses.

Finally, despite the disorders looking different “on paper,” clinicians may often find the diagnostic landscape challenging when looking at the clinical presentations. Some features of ASD, such as restricted interests and repetitive behaviors, may present as inattention and hyperactivity, respectively. Given the criteria changes present in the DSM-5, where ADHD and ASD are no longer mutually exclusive diagnoses, another challenge clinicians face is treating individuals with both disorders. These individuals make up a unique group that, to date, has been poorly studied. Those with comorbid ASD and ADHD have shown worse functioning- showing deficits in communication, social skills, adaptability, and skills of daily living. A comorbid diagnosis can also exacerbate the symptoms of each disorder and result in a worse functioning and quality of life than either ADHD or ASD alone.

## 8 Strengths and limitations

The primary limitation of this review was a lack of strong and consistent research surrounding this topic in the current global fund of knowledge. Since the comorbid diagnosis is a relatively new phenomenon, there is an inherent lag in the public fund of knowledge. Many of the articles reviewed had conflicting results that at times were contradictory with one another. Furthermore, despite the change in diagnostic criteria, there are still relatively few studies that compare the symptoms of ADHD, ASD, and a comorbid diagnosis of ADHD and ASD. This is an inherent limitation due to the period of time in which this was investigated. Another limitation of this study was locating quality articles that allowed for objective comparison between children with ADHD, ASD, comorbid ADHD and ASD, when compared to neurotypical children. The addition of this control group would likely provide useful insights into how children that have the comorbid diagnosis truly compare to neurotypical children in a measured way. The strengths in this review are that it shows an adequate and up-to-date understanding of what the current knowledge base holds.

## 9 Future directions

Looking to the future, as more children are diagnosed with both ADHD and ASD, more research will ultimately help improve the lives of individuals with these neurodevelopmental disorders. Specifically, during the review process, several gaps were identified for future exploration. In terms of treatments, more research is needed to determine if a comorbid diagnosis warrants changes in treatment modalities aside from combining pharmacology and non-pharmacological treatments that are classically used in each disorder separately. The potential benefits and risks of combined treatment approaches should be assessed. Now that there is heightened attention to the possibility of a comorbid diagnosis, future longitudinal studies on this topic should be explored. Several ideas include tracking developmental trajectories of children with comorbid diagnoses into adulthood to figure out how symptom presentation changes. In the same line of thought, functional outcomes such as relationships, employment, and rates of independent living should also be investigated in this particular population. Since care for children with these presentations heavily relies on parental support, new investigations on the experiences and obstacles that caregivers face should be created to figure out how best to support families and reduce caregiver stress and burnout. There is also room for possible creation of new screening tests that may aid in disambiguating what conditions the child may truly have. With proper interest and funding, investigating this burgeoning topic of interest can lead to a better understanding of not only the comorbidity, but also enrich the community with a heightened understanding of each separate pathology. It is imperative to closely study how a co-occurring diagnosis changes functioning and clinical manifestations to develop more focused assessments and consider treatment plans carefully. In the clinical setting, clinicians must conduct thorough and multidimensional evaluations to customize treatments effectively. Collaboration between clinicians and researchers is essential to addressing the growing need for enhanced diagnostic accuracy and tailored interventions in this population.

## 10 Conclusions

The overlap between ADHD and ASD poses a significant challenge for clinicians in distinguishing the two conditions when one is present. The DSM-5-TR’s recognition that ADHD and ASD are no longer mutually exclusive diagnoses has led to increased comorbid diagnoses, highlighting the need for more research in this area. Both disorders share deficits in executive function, social function, and emotional intelligence, with co-occurring ADHD and ASD often leading to worse executive function. Social dysfunction and difficulties with emotion regulation are also prevalent in both disorders and are intensified in those with co-occurring diagnoses. Lastly, a comorbid ADHD and ASD diagnosis is recognized to be more severe for the individual, leading to a lower quality of life than either disorder alone. These realities emphasize the importance of continued research and understanding of comorbid ADHD and ASD to improve diagnosis and treatment for individuals who exhibit overlapping symptoms.
